# Intraoperative ventilation strategies for obese patients undergoing bariatric surgery: systematic review and meta-analysis

**DOI:** 10.1186/s12871-020-0936-y

**Published:** 2020-02-04

**Authors:** George Márcio Costa Souza, Gianni Mara Santos, Sandra Adriana Zimpel, Tamara Melnik

**Affiliations:** 1 Universidade Estadual de Ciências da Saúde de Alagoas - Uncisal. Pró-Reitoria de Ensino e Graduação, Rua Jorge de Lima, N 113, Trapiche, Maceió-Al, Maceio, Alagoas 57010-382 Brazil; 2 Universidade Federal de São Paulo - Unifesp, Pró Reitoria de Pós-Graduação, Rua Botucatu, 740 3° andar Sala 305, Vila Clementino, Sao Paulo, Brazil; 3 Universidade Federal de São Paulo - Unifesp. Programa de Pos-graduacao em Saude Baseada em Evidências, Rua Botucatu, 740 3° andar Sala 305, Vila Clementino, Sao Paulo, SP 04023-900 Brazil

**Keywords:** Systematic review, Mechanical ventilation, Obesity, Meta-analysis

## Abstract

**Background:**

Obesity is a global epidemic, and it is widely known that increased Body mass index (BMI) is associated with alterations in respiratory mechanics. Bariatric surgery is established as an effective treatment for this condition.

**Objective:**

To assess the safety and effectiveness of different ventilation strategies in obese patients undergoing bariatric surgery.

**Methods:**

A systematic review of randomized clinical trials aimed at evaluating ventilation strategies for obese patients was carried out. Primary outcomes: in-hospital mortality, adequacy of gas exchange, and respiration mechanics alterations.

**Results:**

Fourteen clinical trials with 574 participants were included. When recruitment maneuvers (RM) vs Positive end-expiratory pressure (PEEP) were compared, RM resulted in better oxygenation *p* = 0.03 (MD 79.93), higher plateau pressure *p* < 0.00001 (MD 7.30), higher mean airway pressure p < 0.00001 (MD 6.61), and higher compliance p < 0.00001 (MD 21.00); when comparing RM + Zero end-expiratory pressure (ZEEP) vs RM + PEEP 5 or 10 cmH2O, RM associated with PEEP led to better oxygenation *p* = 0.001 (MD 167.00); when comparing Continuous Positive Airway Pressure (CPAP) 40 cmH2O + PEEP 10 cmH2O vs CPAP 40 cmH2O + PEEP 15 cmH2O, CPAP 40 + PEEP 15 achieved better gas exchange *p* = 0.003 (MD 36.00) and compliance *p* = 0.0003 (MD 3.00).

**Conclusion:**

There is some evidence that the alveolar recruitment maneuvers associated with PEEP lead to better oxygenation and higher compliance. There is no evidence of differences between pressure control ventilation (PCV) and Volume control ventilation (VCV).

## Background

Obesity is a global epidemic that causes major economic, social and psychological impacts [1]. Body mass index (BMI) values above 30 Kg/m [2] can result in a reduction in life expectancy similar to that caused by smoking [2, 3]. Bariatric surgery is an effective intervention against weight gain and the majority of people who undergo such surgery show an improvement in, or the resolution of, conditions such as diabetes, dyslipidemia, hypertension and obstructive sleep apnaea [4].

The growing number of bariatric surgeries highlights the importance of invasive ventilator support. Anesthetic induction in obese patients can result in a significant reduction in respiratory compliance and increase resistance and pressure in the airway [5]. A correlation has also been found between a high BMI and an increase in breathing effort and a reduction in oxygenation levels, which may lead to atelectasis and slower weaning from mechanical ventilation [6, 7].

To date, no standard ventilation strategy has been established for obese patients, although there is some evidence that recruitment maneuvers (RM) combined with Positive End-Expiratory Pressure (PEEP) improves oxygenation and compliance in comparison with other strategies [8]. A systematic review can therefore make a significant contribution to the decision-making process of healthcare professionals, particularly surgeons and anesthesiologists, when choosing the best ventilation strategy during the surgery and anesthesia of obese patients, with the aim of reducing complications, costs and mortality.

### Objectives

To assess the effectiveness and safety of different ventilation strategies for obese participants undergoing bariatric surgery under general anesthesia.

## Methods

The methodology described by the Cochrane Collaboration was employed in this systematic review [9].

This research was approved by the ethics committee of the federal university of São Paulo - Unifesp - CAAE: 57099216.0.0000.5505.

### Criteria for considering studies for this review

Randomized controlled trials (RCTs) that evaluated different ventilation strategies for obese patients undergoing bariatric surgery, under general anesthesia, regardless of age and gender, were included.

Obesity was defined as BMI greater than 30 Kg/m^2^ [10].

Primary outcomes: in hospital mortality, adequacy of intra-operative gas exchange, pulmonary mechanics (plateau pressure, mean airway pressures, lung compliance and lung resistance) alteration.

Secondary outcomes: Intraoperative and postoperative respiratory complications such barotrauma, hemodynamic instability, pneumonia, atelectasis, reintubation, self-extubation and the need for noninvasive mechanical ventilation measured in hours or days; cardiovascular responses; need for hospitalization in the intensive care unit (ICU) and length of stay (LOS) in the post-anesthesia care unit (PACU).

### Search methods for identification of studies

Searches (see attachment) were performed in the Cochrane Central Register of Controlled Trials; MEDLINE via Ovid (1966 to present); old MEDLINE (1951 to present); and EMBASE via Ovid (January 1990 to present), without language or location restrictions. The highly sensitive Cochrane filter for randomized controlled trials was applied to the MEDLINE and EMBASE searches. Trial registers such as www.clinicaltrials.gov and the Current Controlled Clinical Trials Website (http://www.controlled-trials.com/) were also searched for ongoing trials.

### Data collection and analysis

Two authors (GMCS and SAZ) independently screened all the potential studies identified and coded them as ‘retrieve’ (eligible or potentially eligible/unclear) or ‘do not retrieve’. The full-text reports/publications were then retrieved and two authors independently screened the full text and identified the studies for inclusion. Disagreements were resolved through discussion or if required consultation with a third author. Duplicates were excluded and multiple reports of the same study were collated so that each study, rather than report, is the unit of interest in the review. The selection process was recorded in appropriate detail, as set out in the complete PRISMA flow diagram [11].

The authors were contacted and additional details were requested. Disagreements were resolved by consensus or by involving a third author.

### Assessment of risk of bias in the included studies

Risk of bias was assessed at study level using Cochrane’s ‘Risk of Bias’ tool [12]. Two review authors (GMCS and SAZ) independently assessed the methodologic quality of each study included and resolved their disagreements by discussion.

### Data synthesis

To consider the measures of treatment effect for dichotomous outcomes, the total number of events within each randomized group were entered and the risk ratios with 95% confidence intervals (CI) were calculated. For data presented in other forms, such as odds or hazard ratios, the generic variance option was used, although different effect measures (odds, risk or hazard ratios) were not combined in the same model. Mean differences were calculated for continuous outcomes measured on the same scale in different studies.

### Assessment of heterogeneity

Statistical heterogeneity was evaluated by assessing forest plots and examining the I^2^ value, which describes the proportion of total variation across studies caused by heterogeneity rather than chance [9]. An I^2^ value greater than 50% was considered as the cut-off point to identify the presence of considerable heterogeneity [9].

## Results

**Fig. 1:**
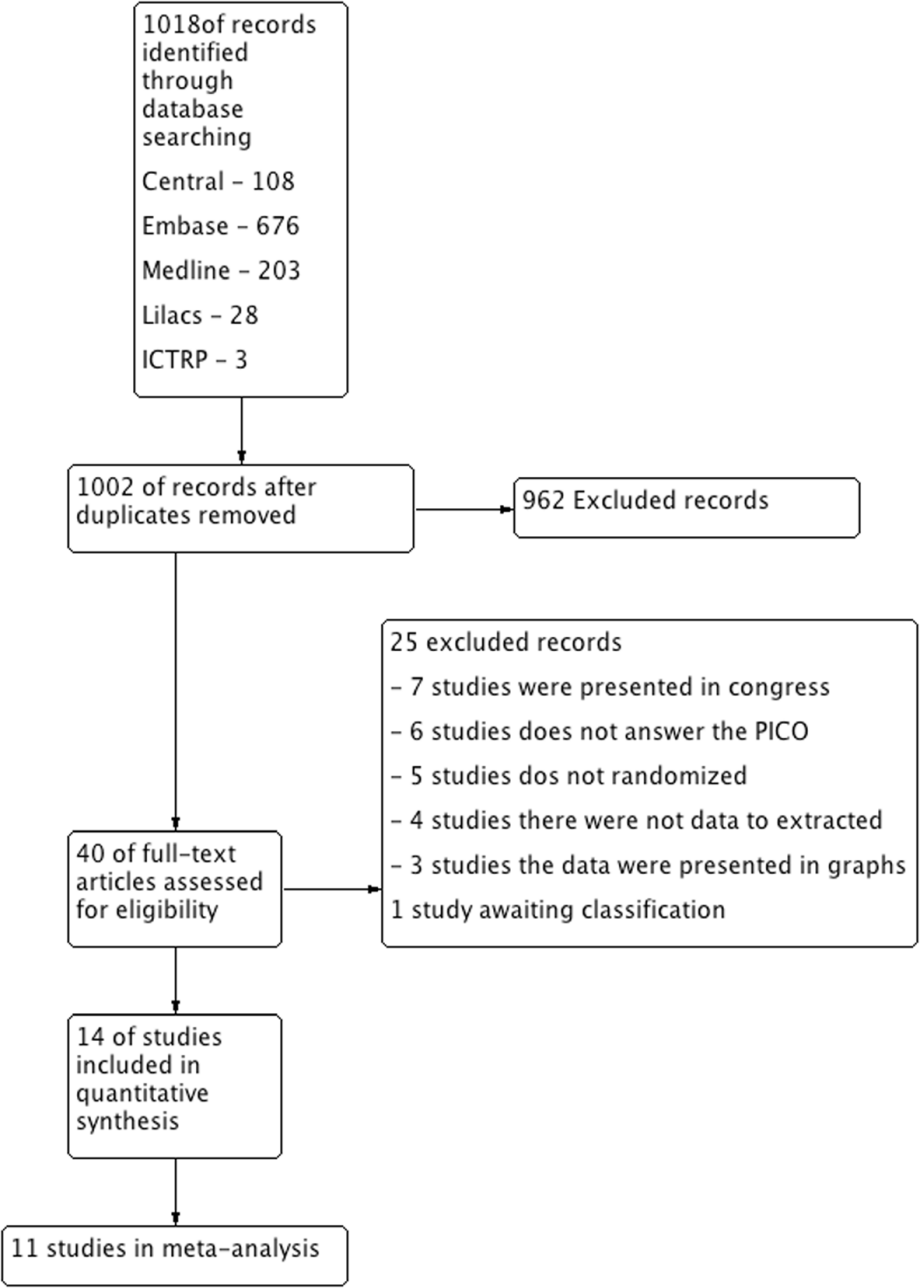
Study flow diagram

The initial search identified 1018 citations through database searches and manual searches (Fig. 1). After screening by title and abstract, full-text articles of 40 studies that were potentially eligible for inclusion in the review were obtained. A total of 25 of these were excluded due to not being randomized, presenting data in graphs, did not present data for extraction or did not respond to the PICO of this review. Following this process, fourteen studies were included in the review (Table 1).

**Table 1:** Characteristics of included studies

Study	Sample	Ventilation strategies	Intervention	Outcomes of interest
Baltieri 2015 [13]	*n* = 40, BMI between 40 and 55 kg m-2	Mode VCV, FiO_2_ between 40 and 60%, Vt = 6–8 ml/kg, PEEP = 5 cmH_2_O (except the G-intra)	Pré-group (*n* = 10): NPPV before surgery for 1 h	Length of stay in PACU
			Intra group (*n* = 10): PEEP = 10 cmH_2_O throughout the surgery.	
Cadi 2008 [14]	*n* = 36, BMI > 35 kg m-2	VCV: VC = 8 ml/kg; RR = 14 irpm; I:E = 1:2; FiO_2_ = 60%; PEEP = 5 cmH_2_O and ispiratory pressure to keep VC = 8 ml/kg	PCV (*n* = 18): I:E = 1:2; FiO_2_ = 60%; PEEP = 5 cmH_2_O and inspiratory pressure to keep VC = 8 ml/kg	Gas exchange PaO_2_/FiO_2_;
				Pulmonary mechanics;
				Ccardiovascular responses
Chalhoub 2007 [15]	*n* = 52, BMI > 40 kg m-2	VCV: VC = 10 ml/kg; I:E = 1:4; FiO_2_ = 40%; RR to keep EtCO_2_ between 30 and 35 mmHg	Group 2 (*n* = 26): ARM (VCM) plus PEEP = 8 cmH_2_O.	Cardiovascular responses
			VCM with pressure of 40 cmH_2_O for 15 s	
De Baerdemaeker 2008 [16]	*n* = 24, BMI > 35 kg m-2	VCV: VC = 10 ml/kg, RR =12 irpm, I:E = 1:2, PEEP = 5 cmH_2_O e FiO_2_ = 50%	PCV (*n* = 12): inspiratory pressure to keep VC = 10 ml/kg and limited at 35 cmH_2_O, EtCO_2_ between 35 and 40 mmHg, PEEP = 5 cmH_2_O	Pulmonary mechanics;
				Cardiovascular responses
Defresne 2014 [17]	*n* = 50, BMI > 35 kg m-2	VCV: VC = 6 ml/kg; PEEP = 10 cmH_2_O e RR to keep Etco_2_ between 4,7 e 6 Kpa.	MR (*n* = 25): inspiratory pressure = 40 mmHg for 40s twice, 5 min after the pneumoperitoneum and 5 min after the pneumoperitoneum plus PEEP = 10 cmH_2_O	Pulmonary mechanics
El-Sayed 2012 [18]	*n* = 56, BMI > 50 kg m-2	Group 1: VCV, FiO_2_ = 50%; vc = 8–10 ml/kg; I:E = 1:2; PEEP = 0 cmH_2_O; RR to keep EtCO_2_ between 30 and 35 mmHg;	Group 2(*n* = 19): VCM of 40 cmH_2_O for 15 s plus PEEP = 15 cmH_2_O.	Gas exchange PaO_2_/FiO_2_
			Group 3 (n = 18): VCM of 40 cmH_2_O for 15 s plus PEEP = 15 cmH_2_O plus NPPV 12/8.	Pulmonary mechanics
				Cardiovascular responses
				Intraoperative and postoperative Respiratory complications
Futier E 2011 [19]	*n* = 66, BMI > 40 kg m-2	Group 1: VCV, VC = 8 ml/kg; RR to keep PaO_2_ between 35 and 42 mmHg, I:E = 1:2; PEEP = 10 cmH_2_O e FiO_2_ = 50%	Group 2(*n* = 22): NPPV	Cardiovascular responses
			Group 3 (n = 22): NPPV plus ARM (after intubation)	
			ARM with VCM of 40 cm H_2_O during 40s.	
Mousa 2013 [20]	*n* = 30, BMI > 40 kg m-2	PCV: FiO_2_ = 50%; PEEP = 5 cmH_2_O; inspiratory pressure to keep VC with 8 ml/kg; RR to keep EtCO_2_ between 35 and 40 mmHg	I:E (*n* = 15) = 1:1	Pulmonary mechanics
				Cardiovascular responses
Reinius 2009 [21]	n = 30, BMI > 40 kg m-2	VCV: FiO_2_ = 50%; PEEP = 0 cmH_2_O; VC = 10 ml/kg; RR = 12 irpm ou EtCO_2_ between 34 and 41 mmHg; I:E = 1:2	- Group ARM plus ZEEP (*n* = 10)	Gas exchange PaO_2_/FiO_2_
			- Group MR plus PEEP = 10 cmH_2_O (n = 10)	Pulmonary mechanics
			ARM was performed with inspiratory pressure = 55 cmH_2_O plus inspiratory hold of 10s,	Cardiovascular responses
Remístico P 2011 [22]	n = 30, BMI 35,6 kg m-2	VCV: Details not described in the article	Experimental group: (*n* = 15): ARM with PEEP of 30 cmH_2_O plus inspiratory pressure of 15 cmH_2_O above PEEP, for 2 min, after pneumoperitoneum.	Cardiovascular responses
Souza 2009 [23]	*n* = 47, BMI > 40 kg m-2	VCV: VC = 8–10 ml/kg; FiO_2_ = 50%; PEEP = 5 cmH_2_O e FR between 12 and 14 irpm.	Group ARM 10, 15 and 20 (*n* = 17): progressive increase of PEEP to 10, 15 and 20 cmH_2_O plus 40s inspiratory hold in each step for 2 min.	Gas exchange PaO_2_/FiO_2_
				Pulmonary mechanics
			Group ARM 30 (*n* = 16): PEEP of 30 cmH_2_O for 2 min plus inspiratory hold of 40s.	
			After the ARM the PEEP was keep in 5 cmH_2_O.	
Sprung 2009 [24]	n = 20, BMI = > 40 kg m-2	VCV: RR = 8 irmp (or to keep EtCO_2_ between 40 and 45 mmHg); VC = 8 ml/kg; PEEP = 4 cmH_2_O; I:E = 1:2; FiO_2_ = 50%	ARM (*n* = 8): progressive increase of PEEP to 4, 10, 15 for three cycles and after more 20 cmH_2_O of PEEP for 10 cycles, after the ARM the PEEP was keep in 12 cmH_2_O	Gas exchange PaO_2_/FiO_2_
				Pulmonary mechanics
Talab 2009 [7]	*n* = 58, BMI between 30 and 50 kg m-2	VCV: FiO_2_ = 50%; VC between 8 and 10 ml/kg; RR to keep EtCO_2_ between 32 and 36 mmHg;	Group VCM plus ZEEP (*n* = 19)	Cardiovascular responses
			Group VCM plus PEEP = 10 cmH_2_O (*n* = 20)	Intraoperative and postoperative Respiratory complications
			VCM was apply for 7–8 s after intubation.	Length of stay in PACU
			The details of the VCM were not described	
Whalen 2006 [25]	n = 20, BMI > 40 kg m-2	VCV: FiO_2_ = 50%, RR = 8 irpm; VC = 8 ml/kg; PEEP = 4 cmH_2_O; I:E = 1:2.	ARM (n = 10): progressive increase of PEEP to 4, 10, 15 for three cycles and after more 20 cmH_2_O of PEEP for 10 cycles.	Pulmonary mechanics
				Intraoperative and postoperative Respiratory complications
			The number of ARM depended of the PaO_2_. After ARM the PEEP was keep in 12 cmH_2_O.	

Legends: BMI: Body Mass Index; NPPV: noninvasive positive pressure ventilation; VCM: vital capacity maneuvers; VCV: volume-controlled ventilation; PCV: Pressure-controlled ventilation; FiO_2_: inspired fraction of oxygen; RR: respiratory rate; V_t_: tidal volume; PEEP: positive end-expiratory pressure; ZEEP: zero end-expiratory pressure; I:E: inspiratory-to-expiratory ratio; ARM: alveolar recruitment maneuvers; ETCO_2_: end-tidal CO_2_; VCM: viral capacity maneuver; PIP: peak inspiratory pressure

### Risk of bias in included studies

**Fig. 2:**
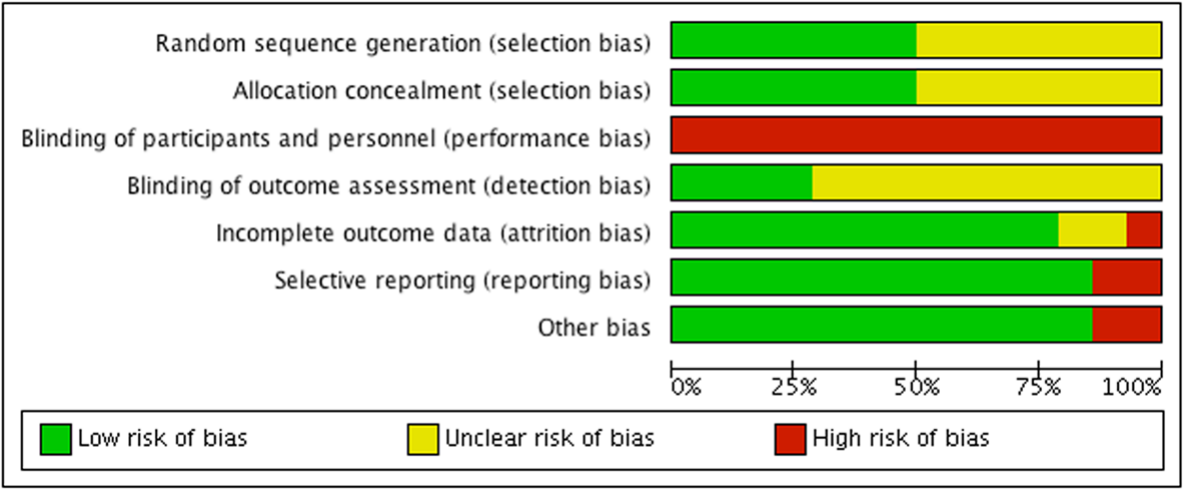
Risk of bias graph: review authors’ judgements about each risk of bias item presented as percentages across all included studies

Random sequence generation and allocation concealment were correctly described in seven studies [14, 17, 19, 20, 22, 24, 25]. In the blinding of participants and personnel domain all the studies were classified as high risk as the personnel could not be blinded. Four studies [7, 13, 17, 25] adequately described the blinding of outcome assessment. Eleven studies [7, 14–21, 24, 25] did not describe losses or exclusions which could cause imbalance between the groups. Only two studies [17, 21] employed selective reporting and while two studies [17, 25] presented other sources of bias Fig. 2.

### Effects of interventions

#### Alveolar recruitment maneuvers versus PEEP only

**Fig. 3:**
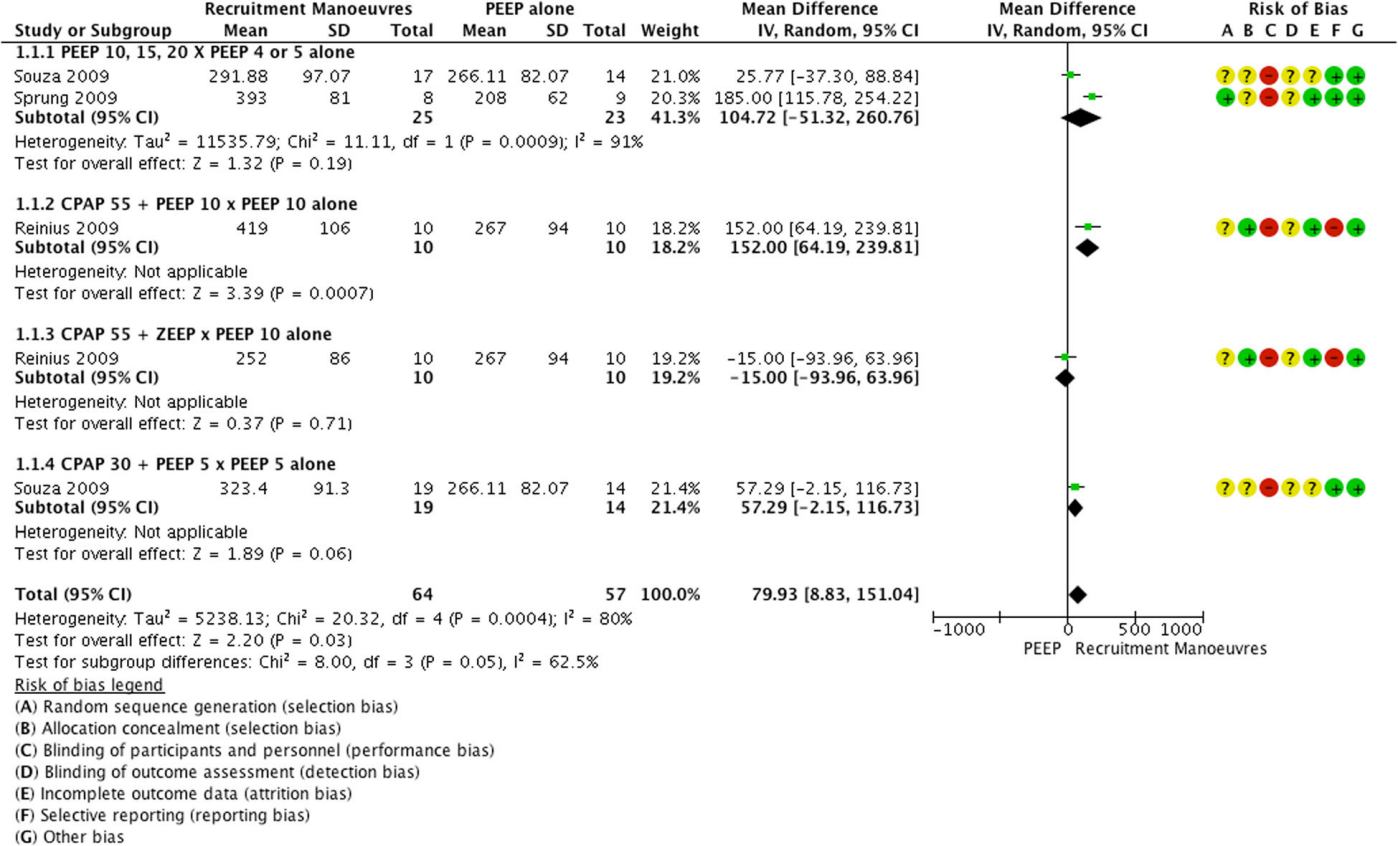
Forest plot of recruitment manoeuvres x PEEP: Intra operative gas exchange - PaO_2_/FiO_2_

Three studies [21, 23, 24] compared alveolar recruitment maneuvers (RM) versus PEEP to evaluate intra operative gas exchange, with the mean PaO_2_/FiO_2_ ratio found to be greater in the groups that underwent RM, *p* = 0.03, (MD 79.93, 95% CI 8.83 to 151.04; participants = 121; studies = 5; I^2^ = 80%,). Figure 3 shows the comparison of three different studies, separate in four subgroups, where the best results were in favor of RM Fig. 3.

**Fig. 4:**
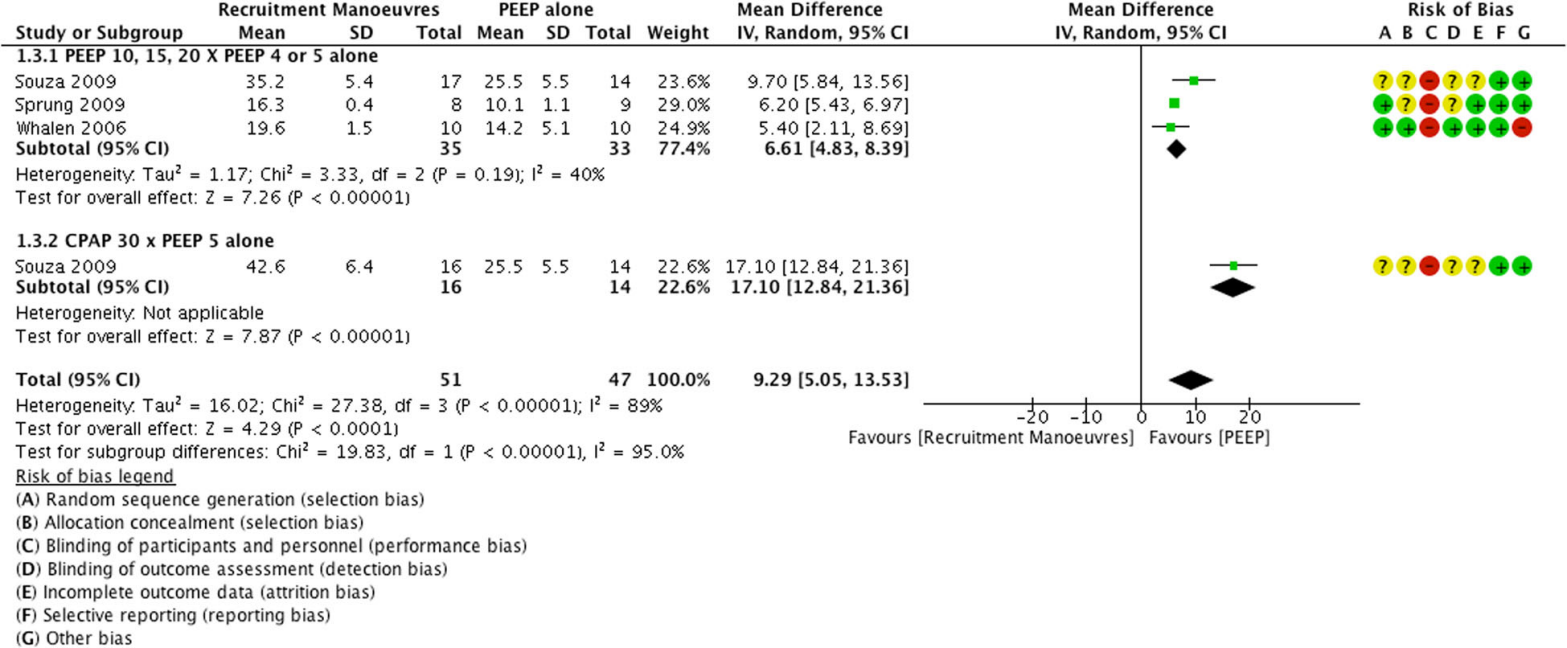
Forest plot of recruitment manoeuvres x PEEP: Mean airway pressure (cmH_2_O)

Three studies [23–25] evaluated mean airway pressures by comparing RM with progressive PEEP of 10, 15 and 20 cmH_2_O versus Peep of 4 or 5 cmH_2_O only and found that the use of PEEP without RM led to lower airway pressure, *p* < 0.001 (MD 9.29, 95% CI 5.05 to 13.53; participants = 98; studies = 4; I^2^ = 89%). Figure 4 shows the comparison of three different studies, separate in two subgroups, where the best results were in favor of PEEP when airway pressure was measured. Figure 4.

**Fig. 5:**
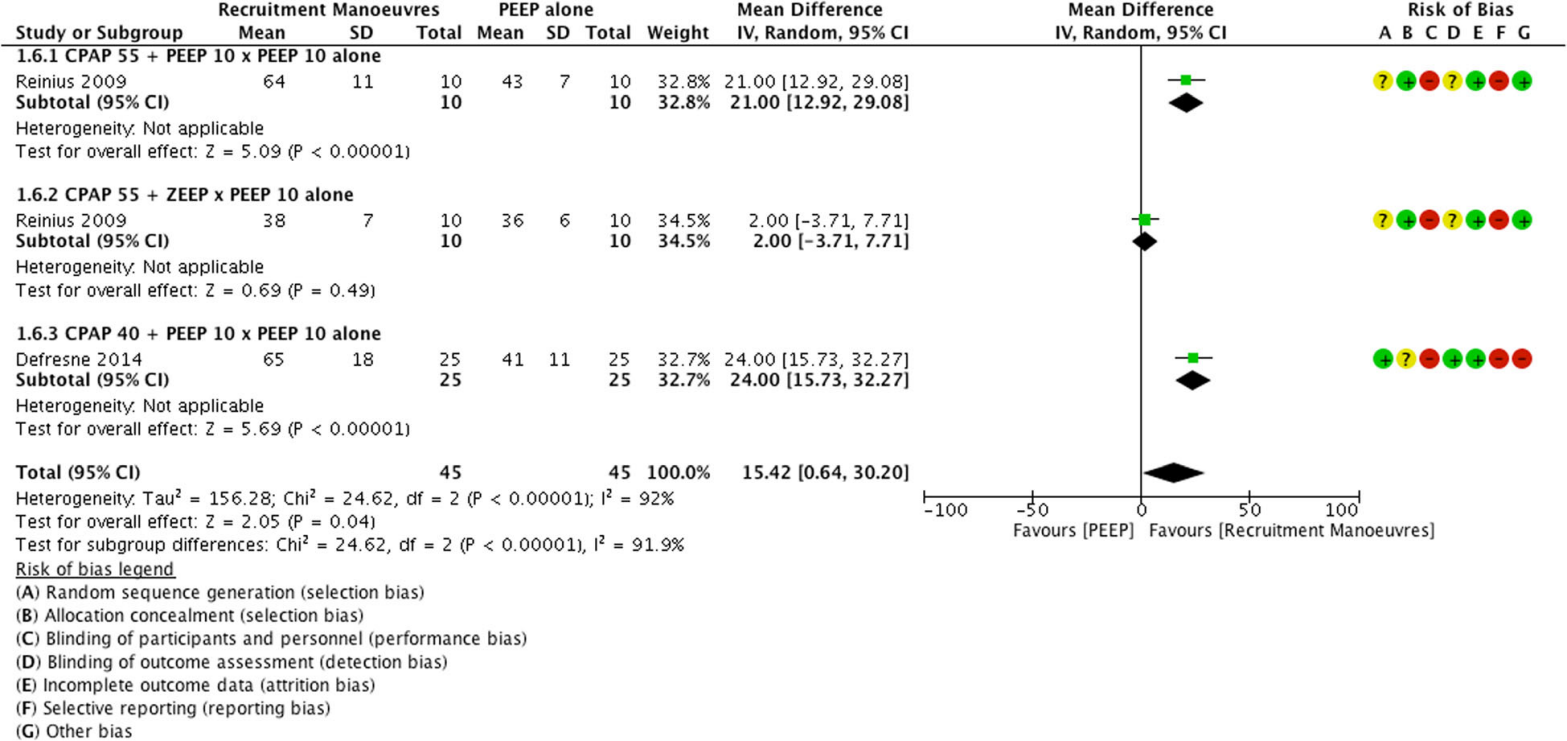
Forest plot of recruitment manoeuvres x PEEP: Compliance (ml cmH_2_O^− 1^)

Two studies [17, 21] evaluated compliance by comparing RM with PEEP. The study by Reinius et al. [21] compared RM with 55 cmH_2_O CPAP plus 10 cmH_2_O PEEP versus 10 cmH_2_O of PEEP only, and identified greater compliance when using RM plus PEEP, *p* < 0.00001 (MD 21.00, 95% CI 12.92 to 29.08; participants = 20; studies = 1; I^2^ = 0%). The same study by Reinius et al. [21] also compared RM with CPAP 55 cmH_2_O plus ZEEP versus PEEP with 10 cmH_2_O, only and found no difference between the groups for compliance, *p* = 0.49 (MD 2.00, 95% CI − 3.71 to 7.71; participants = 20; studies = 1; I^2^ = 0%). The study by Defresne et al. [17] compared RM with CPAP of 40 cmH_2_O plus PEEP 10 cmH_2_O versus 10 cmH_2_O of PEEP only and found greater compliance when using RM plus PEEP, *P* < 0.00001 (MD 24.00, 95% CI 15.73 to 32.27; participants = 50; studies = 1; I^2^ = 0%). When all the studies were taken together, the groups receiving RM exhibited better pulmonary compliance, *p* < 0.04, (MD 15.42, 95% CI 0.64 to 30.20; participants = 90; studies = 3; I^2^ = 92%,). Figure 5 shows the comparison of two different studies, separate in three subgroups, where the best results were in favor of RM when pulmonary compliance was measured. Figure 5.

Four studies [15, 19, 21, 22] evaluated mean arterial pressure. The studies by Chalhoub et al. [15]; Futier et al. [19] and Reinius et al. [21] compared RM with CPAP of 40 or 55 cmH_2_O versus 8 or 10 cmH_2_O of PEEP only and found no difference between the groups, *p* = 0,84, (MD 0.87, 95% CI − 3.77 to 5.52; participants = 116; studies = 3; I^2^ = 0%). The study by Remístico et al. [22] compared a group with RM combined with inspiratory pressure of 15 cmH_2_O and 30 cmH_2_O of PEEP versus PEEP of 5 cmH_2_O only, and found no difference between the mean arterial pressure of the groups, *p* = 0.41, (MD 4.00, 95% CI − 5.45 to 13.45; participants = 30; studies = 1; I^2^ = 0%). The study by Reinius et al. [21] compared a group receiving RM with CPAP of 55 cmH_2_O plus ZEEP versus 10 cmH_2_O PEEP only, and also found no difference between the mean arterial pressure of the groups, *p* = 0.74, (MD 2.00, 95% CI − 9.69 to 13.69; participants = 20; studies = 1; I^2^ = 0%). When all the studies were grouped there was no difference in mean arterial pressure between RM versus the use of PEEP without RM, *p* = 0.44, (MD 1.54, 95% CI − 2.39 to 5.47; participants = 166; studies = 5; I^2^ = 0%).

#### Pressure control ventilation versus volume control ventilation

Of the two included studies that evaluated this comparison [14, 16], only the study by Cadi et al. [14] evaluated the PaO_2_/FiO_2_ ratio, finding that the PCV mode achieved greater oxygenation than the VCV mode, *p* = 0.007, (MD 82.00, 95% CI 21.90 to 142.10; participants = 36; studies = 1; I2 = 0%). The study by De Baerdemaeker et al. [16] did not identify differences in the variables analyzed.

No differences were found between the VCV and PCV modes in the evaluation of mean airway pressure, plateau pressure, lung compliance, lung resistance and arterial pressure.

#### Alveolar recruitment maneuver plus ZEEP versus the same RM plus 5 or 10 cmH_2_O of PEEP

The study by Reinius et al. [21] compared RM with 55 cmH_2_O CPAP plus ZEEP versus the same RM plus 10 cmH_2_O of PEEP and found an improvement in oxygenation in the group with RM plus PEEP 10 cmH_2_O *p* = 0.001, (MD 167.00, 95% CI 82.40 to 251.60; participants = 20; studies = 1; I^2^ = 0%) and in compliance *p* < 0.00001, (MD 26.00, 95% CI 17.92 to 34.08; participants = 20; studies = 1; I^2^ = 0%) when PEEP of 10 cmH_2_O was used.

The study by Talab et al. [7] evaluated the length of stay (LOS) in the post- anesthesia care unit (PACU), comparing the RM with CPAP 40 cmH_2_O plus ZEEP versus RM with CPAP 40 cmH_2_O followed by PEEP 10 cmH_2_O and found a shorter LOS in the PACU in the RM plus PEEP group, *p* = 0.02, (MD -21.05, 95% CI − 38.90 to − 3.20; participants = 39; studies = 1; I^2^ = 0%), The same authors compared RM with 40 cmH_2_O CPAP plus ZEEP versus ARM with 40 cmH_2_O CPAP plus 5 cmH_2_O PEEP and found no difference between the LOS in PACU of the groups, *p* = 0.26, (MD -10.45, 95% CI − 28.78 to 7.88; participants = 38; studies = 1; I^2^ = 0%). When the two comparisons were pooled the shortest LOS in the PACU was found in the group that received RM plus PEEP, *p* = 0.01, (MD -15.89, 95% CI − 28.68 to − 3.10; participants = 77; studies = 2; I^2^ = 0%).

The study by Talab et al. [7] compared RM with CPAP 40 cmH_2_O plus ZEEP versus RM with CPAP 40 cmH_2_O plus PEEP 10 cmH_2_O and found fewer patients with lamellar atelectasis in the group that received RM plus ZEEP, *p* = 0.007, (RR 5.22, 95% CI 1.33 to 20.55; participants = 39; studies = 1; I^2^ = 0%). The same author Talab et al. [7] compared RM with CPAP 40 cmH_2_O plus ZEEP versus RM with CPAP 40 cmH_2_O plus PEEP 5 cmH_2_O and found no difference in lamellar atelectasis between the groups, *p* = 0.38, (RR 2.00, 95% CI 0.41 to 9.65; participants = 38; studies = 1; I^2^ = 0%). When the two comparisons were pooled there was a smaller proportion of lamellar atelectasis in the group that underwent RM plus ZEEP, *p* = 0.03, (RR 3.45, 95% CI 1.23 to 9.71; participants = 77; studies = 2; I^2^ = 0%). When Talab et al. [7] evaluated RM with CPAP 40 cmH_2_O plus ZEEP versus RM with CPAP 40 cmH_2_O plus PEEP 10 cmH_2_O, fewer patients with segmental atelectasis were found in the group that received RM plus 10 cmH_2_O PEEP, *p* = 0.009, (RR 0.29, 95% CI 0.12 to 0.74; participants = 39; studies = 1; I^2^ = 0%).

Talab et al. [7] also compared RM with CPAP 40 cmH_2_O plus PEEP 5 cmH_2_O versus RM with CPAP 40 cmH_2_O plus PEEP 10 cmH_2_O and found a difference between the groups, with a lower number of patients with Lamellar atelectasis in the RM plus PEEP 5 cmH_2_O group, *p* = 0.05, (RR 2.61, 95% CI 1.00 to 6.80; participants = 39; studies = 1; I^2^ = 0%).

#### CPAP 40 plus PEEP10 versus CPAP 40 plus PEEP15

El-Sayed et al. [18] compared CPAP 40 cmH_2_O plus PEEP 10 cmH_2_O versus CPAP 40 cmH_2_O plus PEEP 15 cmH_2_O and found that CPAP plus PEEP 15 cmH_2_O achieved a greater PaO_2_/FiO_2_ ratio, *p* = 0.003, (MD 36.00, 95% CI 12.10 to 59.90; participants = 38; studies = 1; I^2^ = 0%) and greater lung compliance cmH_2_O, *p* = 0.0003, (MD 3.00, 95% CI 1.38 to 4.62; participants = 38; studies = 1; I^2^ = 0%).

#### Alveolar recruitment maneuver plus PEEP 10, 15 and 20 versus CPAP 30

The study by Souza et al. [23] compared the use of RM with progressive PEEP of 10, 15 and 20 cmH_2_O versus CPAP 30 cmH_2_O and found that the RM with progressive PEEP obtained lower mean airway pressures, p = 0.0003, (MD -7.40, 95% CI − 11.45 to − 3.35; participants = 33; studies = 1; I^2^ = 0%).

#### PEEP 10 versus PEEP 5

The study by Baltieri et al. [13] compared the use of PEEP 10 cmH_2_O versus PEEP 5 cmH_2_O and found no difference between the groups in LOS in the PACU, *p* = 0.21, (MD 36.00, 95% CI − 20.16 to 92.16; participants = 30; studies = 1; I^2^ = 0%).

#### I:E 1:1 ratio versus I:E 1:2 ratio

The study by Mousa et al. [20] evaluated the I:E 1:1 ratio versus the I:E 1:2 ratio and found that the I:E 1:1 ratio group achieved greater lung compliance than the group with a I:E 1:2 ratio, *p* = 0.01, (MD 4.67, 95% CI 1.06 to 8.28; participants = 30; studies = 1; I^2^ = 0%, low-quality evidence).

## Discussion

The present systematic review evaluated different ventilatory strategies for obese patients undergoing bariatric surgery, such as: comparison between PCV and VCV; comparison of different forms of RM, different PEEP levels and comparison between I:E 1:1 ratio and I:E 2:2. Fourteen studies with a total of 574 participants were included.

Significant variability in interventions were found. This demonstrates the lack of consensus on how to ventilate obese patients undergoing surgery, corroborating a review published by Aldenkortt et al. [8]

The main finding of the present study is the evidence that obese patients receiving mechanical ventilation benefit from RM, especially when combined with PEEP, as evidenced by improvements in oxygenation and respiratory compliance. While it was observed in this systematic review that the isolated use of PEEP was more effective when higher values were used, however the best result was the combination of the RM with higher levels of PEEP. In addition to these findings, no difference was found between VCV and PCV modes of ventilation in all analyzed outcomes, corroborating another study by Aldenkortt et al. [8]. No respiratory complications or major adverse events were reported in the studies included in this review. Such findings are similar to those found by Aldenkortt et al. [8]. and Hu et al. [26]. Recent guidelines regarding mechanical ventilation of patients with acute respiratory distress syndrome (ARDS) have shown that the incidence of complications associated with diferent mechanical ventilation strategies is low [27].

There is insufficient evidence to support differences between VCV and PCV in the evaluated outcomes. While the study by Cadi et al. [14]. Showed that the pressure controlled mode led to a higher PaO_2_/FiO_2_ ratio than the volume controlled mode, the study by De Baerdemaeker et al. [16]. Did not identify a difference in PaO_2_ between the two modes.

Three studies [17, 24, 25] included in this review describe the performance of more than one alveolar recruitment maneuver. There is no consensus on the ideal number of alveolar recruitment maneuvers regarding frequency and repetitions, however, the use of various maneuvers with patients with ARDS is associated with decreased pulmonary shunt and increased compliance [27].

Despite the wide variety of interventions and outcomes evaluated, the present review provides some evidence that the use of PEEP effectively improves oxygenation and compliance of the respiratory system. Better results seem to be achieved, however, when it is combined with alveolar recruitment maneuvers, and the absence of adverse effects shows that it is an effective and safe strategy for obese patients undergoing bariatric surgery. Briel et al. [28] published a systematic review and meta-analysis comparing the use of high versus low PEEP values for ARDS patients, and concluded that the use of high levels of PEEP was associated with lower hospital mortality in this group of patients. The American Thoracic Society also currently recommends the use of high levels of PEEP for patients with ARDS [27].

One study compared I:E 1:1 ratio with I:E 1:2 ratio and found that only the 1:1 ratio only improved lung compliance [20]. Few studies evaluated the use of the I:E 1:1 ratio, while some studies evaluated different inverted ratios in patients with ARDS, with conflicting results regarding its effectiveness [29–31].

### Limitations

Important methodological limitations of the studies included reduced the certainty of the evidence offered by most of the included trials. Many of the trials were small and included different outcome measures, and selective outcome reporting was occasionally an issue.

The paucity of long-term follow-up data, the small sample sizes, and the heterogeneous nature of the measured outcomes limit the generalizability of the results.

## Conclusions

There is evidence that alveolar recruitment maneuvers plus PEEP improve gas exchange with an increase in respiratory system compliance. The quality of such evidence is low, however.

There is no evidence to support that there is a difference between the volume and pressure controlled modes.

The various interventions assessed were shown to be safe with no major adverse events reported.

## Data Availability

Not applicable. All data from this systematic review were extracted from primary studies. The datasets used and/or analysed during the current study are available from the corresponding author on reasonable request.

## Abbreviations

ARM: Alveolar recruitment maneuvers
BMI: Body mass index
CI: Confidence intervals
CPAP: Continuous Positive Airway Pressure
I:E: Inspiratory:Expiratory ratio
ICU: Intensive care unit
LOS: Length of stay
MD: Mean difference
PACU: Post-anesthesia care unit
PCV: Pressure control ventilation
PEEP: Positive end-expiratory pressure
PICO: Problem, intervention, control, outcome
RCTs: Randomized controlled trials
RM: Recruitment maneuvers
UNIFESP: Universidade Federal de São Paulo
VCV: Volume control ventilation
ZEEP: Zero end-expiratory pressure
